# Law and Prostitution

**Published:** 1882-09

**Authors:** 


### Editorial.

### LAW AND PROSTITUTION.

Ever and anon we find, in various medical journals of this country, the question of legalizing prostitution discussed by advocates for and opponents of the licensing and inspection system, and many are the words and reasons expended on both sides in the agitation of this unsavory subject.

It is a matter concerning the propriety and importance of which the medical profession are supposed to have an opinion, and its final solution—for it bids fair soon to constitute an issue in more than one municipality on this side of the great waters—will doubtless be referred, informally and to a great measure at least, to the medical practitioners of any community in which the problem may be formulated and presented.

It is in anticipation of this impending event that the REGISTER desires to place itself on record and to give the reasons for the faith which is within us.

Thou shalt not commit adultery is as surely written and as plainly commanded as thou shalt not steal. Fornication is as certainly forbidden and as severely condemned as bearing false witness or murder. "Thou shalt not" is the language of the law which is above all other law—nay, it is the decree of Omniscience. What impotent state shall gainsay it? It is the edict of Omnipotence. What puny power shall stay it?

The prohibition of these criminal acts is full, final and unmistakable. It is man's lust, not his necessities; man's carnal desires, not his actual wants, that makes this a living question. Prostitution, notwithstanding the claims that are put forward in its behalf, is not a necessity—it is not a necessary evil. But it is a sin, a shame, an abomination, a great blot upon civilization, a great outrage upon social order. It is the work of the devil, that arch adversary of human happiness and human weal, and pleas for its recognition by law are pleas for the legal degradation of woman and for the deliberate demoralization of man.

The establishment of prostitution by legal enactment as a legitimate vocation would be to array the weakness of man against the supreme law of the universe. It would be an encouragement to crime and would promote a vice the blasting consequences of which would pull down domestic altars, disrupt happy homes, disquiet peaceful communities and sow the seed of physical and moral death broadcast over the land.

We assert that it is no part of the duty of any government, under the deceptive plea of protecting society by the official inspection of prostitutes, and thereby throwing the semblance of security around crime, to encourage the wickedness of the wicked. It is no part of the duty of any government to join hands with the most debased members of society in placing a bondage perpetual, degrading, revolting and destructive upon any class of human beings.

Away with all specious arguments, all ingenious special pleading, and let men know that the law will not interpose to shield them from the natural fruits of criminal acts. Let all transgressors understand that the law of the land is conformed to the requirements of the great moral law under which all the earth shall finally be judged. Let the power of government be directed to the preservation, not to the overthrow of order, and to the full protection of all classes of citizens, always refusing to abandon even those who are reckless of themselves. Always refusing deliberately to deliver over even the lowest representatives of humanity to the living horrors of an eternal death.

Take away fear and then is removed the most potent restraint of the evil passions of men. It will not do. The whole proposition of government protection of criminal, sinning men, and of the moral and physical destruction of weak and wicked women, is based upon a narrow and mistaken view of the entire subject. It is a snare and a delusion. No; in the decision of this question, as in all others, let the right be done though the heavens fall. Let the right, we say, prevail and leave the consequences to take care of themselves.

